# A Ferroptosis-Related Signature Robustly Predicts Clinical Outcomes and Associates With Immune Microenvironment for Thyroid Cancer

**DOI:** 10.3389/fmed.2021.637743

**Published:** 2021-04-13

**Authors:** Mingqin Ge, Jie Niu, Ping Hu, Aihua Tong, Yan Dai, Fangjiang Xu, Fuyuan Li

**Affiliations:** Department of Endocrinology, Linyi Central Hospital, Linyi, China

**Keywords:** ferroptosis, gene signature, thyroid cancer, immune microenvironment, prognosis

## Abstract

**Objective:** This study aimed to construct a prognostic ferroptosis-related signature for thyroid cancer and probe into the association with tumor immune microenvironment.

**Methods:** Based on the expression profiles of ferroptosis-related genes, a LASSO cox regression model was established for thyroid cancer. Kaplan-Meier survival analysis was presented between high and low risk groups. The predictive performance was assessed by ROC. The predictive independency was validated *via* multivariate cox regression analysis and stratified analysis. A nomogram was established and verified by calibration curves. The enriched signaling pathways were predicted *via* GSEA. The association between the signature and immune cell infiltration was analyzed by CIBERSORT. The ferroptosis-related genes were validated in thyroid cancer tissues by immunohistochemistry and RT-qPCR.

**Results:** A ferroptosis-related eight gene model was established for predicting the prognosis of thyroid cancer. Patients with high risk score indicated a poorer prognosis than those with low risk score (*p* = 1.186e-03). The AUCs for 1-, 2-, and 3-year survival were 0.887, 0.890, and 0.840, respectively. Following adjusting other prognostic factors, the model could independently predict the prognosis (*p* = 0.015, HR: 1.870, 95%CI: 1.132–3.090). A nomogram combining the signature and age was constructed. The nomogram-predicted probability of 1-, 3-, and 5-year survival approached the actual survival time. Several ferroptosis-related pathways were enriched in the high-risk group. The signature was distinctly associated with the immune cell infiltration. After validation, the eight genes were abnormally expressed between thyroid cancer and control tissues.

**Conclusion:** Our findings established a prognostic ferroptosis-related signature that was associated with the immune microenvironment for thyroid cancer.

## Introduction

Thyroid cancer is the most often diagnosed endocrine malignancy, accounting for 1% of all newly diagnosed cancers (1). In the past 30 years, the global incidence of thyroid cancer has markedly increased (2). The disease is expected to become the fourth major cancer worldwide (3). Surgery followed by radioactive iodine or observation is the main therapy for most of patients (2). The application of high-throughput technology is increasing, which deepens the understanding about the molecular characteristics of thyroid cancer. Molecular markers have also become effective tools for predicting prognosis and identifying new therapeutic targets in thyroid cancer management.

Activated immune cells in the tumor microenvironment secrete pro-inflammatory cytokines and chemokines, which may promote the progression of thyroid cancer (4). Immunotherapy provides new possibilities for curing thyroid cancer. Recently, the crosstalk between ferroptosis and immune cells is involved in mediating response to the immunotherapy (5). Ferroptosis characterized by unique morphologies (decreased cell size, elevated mitochondrial membrane density) and bioenergy characteristics, is a novel form of regulated cell death with an iron-dependent manner (6). Glutathione depletion or glutathione peroxidase 4 inactivation may lead to the metabolic imbalance, thereby inducing ferroptosis of cancer cells (7). Ferroptosis has exhibited great potential in cancer therapy. FDA has approved small molecule inducers of ferroptosis to kill cancer cells such as Erastin, Sulphasalazine, Sorafenib and Statins (8). However, the exact mechanisms of ferroptosis and the association with the tumor immune microenvironment have not been uncovered. Recently, Liang et al. established a robust prognostic gene signature based on 10 ferroptosis-related genes for hepatocellular carcinoma (9). Furthermore, Wu et al. developed a ferroptosis-related five signature for predicting prognosis for clear cell renal cell carcinoma (10). Nevertheless, at present, there is still a lack of ferroptosis-related gene model for predicting the prognosis of patients with thyroid cancer. Hence, in this study, we established a prognostic ferroptosis-related signature, which could robustly predict the survival time of patients and was associated with the immune microenvironment for thyroid cancer.

## Materials and Methods

### Thyroid Cancer Datasets

RNA sequencing (RNA-seq) transcriptome data of thyroid cancer were downloaded from The Cancer Genome Atlas (TCGA, http://cancergenome.nih.gov/) database on September 14, 2020. Using the Ensembl database (http://asia.ensembl.org/index.html), gene names were transformed from Ensembl ID into gene symbol matrix. The corresponding clinical information including age, gender, stage and TNM was also retrieved from TCGA database. Following removing samples without complete follow-up information, 510 thyroid cancer samples and 58 normal samples were included in this study. Table 1 listed the clinical characteristics of these thyroid cancer patients. Sixty ferroptosis-related genes were obtained from the previous literature (9). The expression profiles of these ferroptosis-related genes were extracted from above samples (Supplementary Table 1). Differentially expressed ferroptosis-related genes were screened between thyroid cancer and normal samples utilizing the edgeR package in R with the threshold of adjusted *p* < 0.05 (11).

**Table 1:** The clinical characteristics of thyroid cancer patients.

**Characteristics**		**Number**
**Age**		
	<65	436
	>65	71
**Gender**		
	Male	136
	Female	371
**Stage**		
	Stage I	285
	Stage II	52
	Stage III	113
	Stage IV	55
	Unknown	2
**T**		
	T1	144
	T2	167
	T3	171
	T4	23
	Tx	2
**M**		
	M0	283
	M1	9
	Mx	213
	Unknown	1

### Construction of a Least Absolute Shrinkage and Selection Operator Cox Model

Differentially expressed ferroptosis-related genes were used for construction of a LASSO Cox model. Prognosis-related genes were evaluated by univariate Cox regression analysis *via* the survival package in R. Genes with *p* < 0.05 were selected for LASSO Cox model analysis. A LASSO Cox model was then established through the Glmnet package in R (12). Variable selection was presented to get better performance parameters, followed by regularization so as to avoid overfitting. The regularization of LASSO was controlled by the parameter λ. The larger the λ, the greater the penalty for linear models with more variables. In this study, a 10-fold cross-validation was presented for each λ and the partial likelihood deviance values was determined. The optimal λ was selected to establish the model. The risk score for each sample was calculated according to β_1_x_1_ + β_2_x_2_ + … + β_p_x_p_ (where β_p_ indicates the coefficients, and x_p_ indicates the gene expression levels). Based on the median value of risk scores, patients were separated into high- and low- risk groups. Kaplan-Meier overall survival (OS) analysis was presented, followed by log-rank test. The sensitivity and accuracy of the signature was validated by the Receiver Operating Characteristic (ROC) curve utilizing the SurvivalROC package in R. Principal Component Analysis (PCA) was presented for assessing the accuracy of the classification according to the high and low risk scores.

### Univariate and Multivariate Cox Regression Analysis

Univariate cox regression analysis was presented for assessment of the prognostic values of the risk score and clinical features (age, gender, stage, T, N, and M). Afterwards, multivariate cox regression analysis was used to determine which prognostic factors could independently predict the survival of patients.

### Nomogram Model Establishment

By combining the eight ferroptosis-related genes, a nomogram was established. Moreover, another nomogram was constructed by combining age and the risk score. The total point of each patient was calculated based on the nomogram. The nomogram-predicted probability of 1-, 3-, and 5-year survival time was contrast by the actual survival time, which was visualized by the calibration curves.

### Stratified Survival Analysis

Patients were divided into different subgroups according to age (<65 and ≥65), gender (female and male) and stage (stage I-II and stage III-IV). Kaplan-Meier survival analysis followed by log-rank test was presented between high and low risk score groups in different subgroups.

### Gene Set Enrichment Analysis

GSEA was performed between high and low risk score groups (13). The “c2.cp.kegg.v7.1.symbols” was utilized as the reference. Signaling pathways with nominal *p*-value < 0.05 and false discovery rate (FDR) <0.05 were significantly enriched.

### Estimation of Immune Infiltration

The infiltration levels of 22 immune cells in thyroid cancer samples were assessed *via* CIBERSORT (http://cibersort.stanford.edu/) (14). The differences in the infiltration levels of immune cells were compared between high and low risk score groups *via* the Wilcoxon rank-sum-test.

### Immunohistochemistry

Immunohistochemistry of DPP4, GPX4, GSS, AKR1C1, HMGCR, TFRC, SQLE, and PGD in thyroid cancer and normal tissues was obtained from The Human Protein Atlas database (https://www.proteinatlas.org/).

### Patients and Specimens

Twenty thyroid cancer and 10 adjacent normal tissues were obtained from Linyi Central Hospital between 2019 and 2020. Thyroid cancer was confirmed by post-operative pathological examination. Before surgery, no patient underwent radiation therapy or thyrotropin suppression therapy. The study was approved by the Ethics Committee of Linyi Central Hospital and followed the Declaration of Helsinki (2019019). Each patient provided written informed consent. All the resected specimens were placed instantly into liquid nitrogen and stored at −80°C.

### Quantitative Real-Time PCR

Total RNA was extracted from tissues or cells *via* TRIzol (Invitrogen, Carlsbad, California, USA), followed by reverse transcription into cDNA. PCR was carried out using the TB Green® Premix Ex Taq™ II kit (TAKARA, China). The reaction procedures were as follows: 40 cycles at 94°C lasting 15 s, 60°C lasting 10 s, and 72°C lasting 20 s. GAPDH served as an internal control. The relative expression levels were quantified with the 2^−ΔΔCt^ method. The primer sequences were listed in Table 2.

**Table 2:** Primer sequences for qRT-PCR.

**Primer**	**Sequence (5^**′**^-3^**′**^)**
DPP4	ATTCCAAACAACACACAGT (forward)
	CTTCATAAACCCAGTCAGT (reverse)
GPX4	GTAAAACCGGACCAGAAGTACAAG (forward)
	CCCACCTGCTTCCCGAACTG (reverse)
GSS	CTGGAGCGGCTGAAGGACA (forward)
	AGCTCTGAGATGCACTGGACA (reverse)
AKR1C1	TGCAGAGGTTCCTAAAAGTAAAGCTTTA (forward)
	GGAAAATGAATAAGGTAGAGGTCAACATAA (reverse)
HMGCR	AGTTTGAAGAGGATGTTTTG (forward)
	TCCCTTACTTCATCCTGTGA (reverse)
TFRC	ACCATTGTCATATACCCGGTTCA (forward)
	CAATAGCCCAAGTAGCCAATCAT (reverse)
SQLE	TGGTTACATGATTCATGATC (forward)
	TACTGAACTCCCATCACAAC (reverse)
PGD	ATTCTCAAGTTCCAAGACACCG (forward)
	GTGGTAAAACAGGGCATGGGA (reverse)
GAPDH	CTGCCCAGAACATCATCC (forward)
	CTCAGATGCCTGCTTCAC (reverse)

### Cell Culture and Transfection

Human thyroid cancer cell lines TPC-1 and FTC-133 (American Type Culture Collection, ATCC) were cultured in Dulbecco's modified eagle medium (Gibco, USA) containing 10% FBS, 100 U/ml penicillin and 100 μg/ml streptomycin in a 5% CO_2_ incubator at 37°C. The cells were seeded in 6-well plates (2 × 10^5^/well). When the cell confluence reached 60%, transfection of siRNA-negative control (si-NC), si-AKR1C1#1 and si-AKR1C1#2 was carried out according to the Lipofectamine 2000 (Invitrogen, USA) instructions. After 48 h, the cells were harvested.

### Flow Cytometry

The transfected cells were seeded in 6-well plates (2 × 10^4^/well). After culturing for 48 h, the culture medium was discarded, and the cells were trypsinized and collected. Following the instructions of Annexin V-FITC / PI kit, 200 μl binding buffer was added to resuspend cells, followed by 5 μl Annexin V-FITC and 5 μl PI. After incubating for 20 min in the dark, the apoptosis rate was detected by flow cytometry (BD, USA).

### Wound Healing

The transfected cells were seeded into 24-well plates (5 × 10^4^/well). The next day, a 200 μl pipette tip was utilized to draw a straight line perpendicular to the bottom of the cell culture plate. The width of scratches (×40) was investigated at 0 and 24 h under a microscope.

### Transwell

Matrigel was diluted at 1:10 by serum-free medium, which was added to the upper layer of the transwell chamber (Corning, USA). The transfected cells diluted to 2 × 10^5^/ml with serum-free medium were added to the upper layer of the chamber at 100 μl per well. Five hundred microliter of medium containing 10% FBS was added to the lower layer. After 24 h, the cells were fixed with formaldehyde and stained with crystal violet. The number of invasive cells was counted.

### Statistical Analysis

Statistical analysis was presented through R 3.6.3 and GraphPad 7.0. Data were presented as means ± standard deviation. Comparisons between two groups were analyzed by paired student's *t*-test or Wilcoxon rank-sum-test. Multiple comparisons were presented by one-way ANOVA. Difference with *p* < 0.05 was considered statistically significant.

## Results

### Differentially Expressed Ferroptosis-Related Genes for Thyroid Cancer

A total of 60 ferroptosis-related genes were included in this study. Among them, 46 genes with adjusted *p* < 0.05 were differentially expressed between 510 thyroid cancer samples and 58 normal samples.

### Establishment of a Prognostic Ferroptosis-Related Eight-Gene Model for Thyroid Cancer

**Figure 1:**
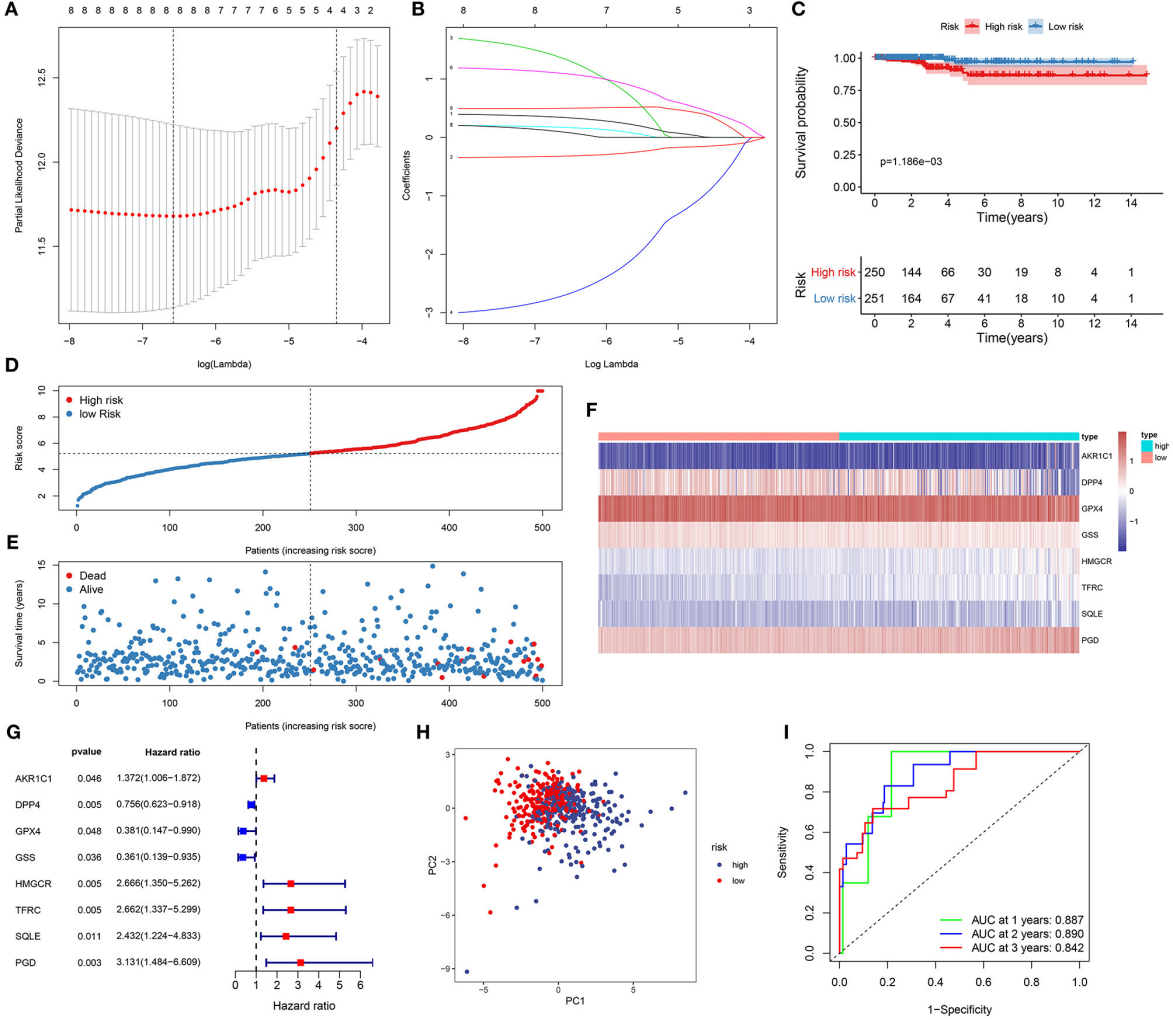
Establishment of a ferroptosis-related eight-gene model for predicting the prognosis of thyroid cancer patients. **(A)** Selection of the optimal λ-value through the 10-fold cross-validation. **(B)** Fitting processes of LASSO Cox regression model. Each curve is indicative of the change trajectory of an independent variable coefficient. The ordinate is the value of the coefficient, the lower abscissa is log(λ), and the upper abscissa is the number of non-zero coefficients in the model. **(C)** Kaplan-Meier overall survival analysis for high (red) and low (blue) risk groups. **(D)** The ranking of the risk scores among all thyroid cancer samples. **(E)** The survival status including dead (red) and alive (blue) in high and low risk groups. **(F)** Heat map visualizing the expression levels of the eight ferroptosis-related genes in high (blue) and low (red) risk groups. **(G)** The prognostic values of the eight ferroptosis-related genes for thyroid cancer based on univariate cox regression analysis. **(H)** PCA for the high (blue) and low (red) risk thyroid cancer samples. **(I)** ROC for 1- (green), 2- (blue) and 3-year (red) survival time for high and low risk patients.

Based on 46 differentially expressed ferroptosis-related genes, prognosis-related genes were selected for LASSO Cox regression analysis. When log λ = −6.6, the model exhibited the optimal performance and the least number of independent variables (Figure 1A). As the values of λ increased, the LASSO coefficients of these variables were close to zero (Figure 1B). As a result, eight ferroptosis-related genes were utilized for the establishment of a prognostic model. The risk score for each sample was calculated as follows: (0.340143834733433) ^*^ AKR1C1 expression + (−0.319305763663027) ^*^ DPP4 expression + (1.3413362890122) ^*^ GPX4 + (−2.69880844893117) ^*^ GSS + (0.171053637326744) ^*^ HMGCR + (1.0923479137719) ^*^ TFRC + (0.0892997114590679) ^*^ SQLE + (0.499540011822522) ^*^ PGD. In line with the median value of all risk scores, 510 thyroid cancer patients were separated into high- and low-risk score groups. Kaplan-Meier curves showed that low risk patients could outlive high risk patients 21 (*p* = 1.186−03; Figure 1C). As the risk scores increased, the number of died patients was gradually increased (Figures 1D,E). Heat map visualized the expression patterns of the eight ferroptosis-related genes between high- and low-risk groups (Figure 1F). Univariate cox regression analysis demonstrated that DPP4 [hazard ratio (HR): 0.756, 95% confidence interval (CI): 0.623–0.918, *p* = 0.004], GPX4 (HR: 0.381, 95% CI: 0.147–0.990, *p* = 0.048) and GSS (HR: 0.361, 95% CI: 0.139–0.935, *p* = 0.036) were protective factors for thyroid cancer prognosis (Figure 1G). Meanwhile, AKR1C1 (HR: 1.372, 95% CI: 1.006–1.872, *p* = 0.046), HMGCR (HR: 2.666, 95% CI: 1.350–5.262, *p* = 0.005), TFRC (HR: 2.662, 95% CI: 1.337–5.299, *p* = 0.005), SQLE (HR: 2.432, 95% CI: 1.224–4.833, *p* = 0.011) and PGD (HR: 3.131, 95% CI: 1.484–6.609, *p* = 0.003) were risk factors for thyroid cancer prognosis (Figure 1G). PCA results confirmed the accuracy of the classification among the thyroid cancer samples (Figure 1H). To further validate the accuracy and sensitivity of the model, we constructed a ROC analysis. Our data confirmed that the model could accurately and sensitively predict the survival probability for 1- [area under the curve (AUC) = 0.887], 2- (AUC = 0.890), and 3-year (AUC = 0.842) survival time (Figure 1I). Collectively, this ferroptosis-related eight-gene model could be a robust prognostic model for thyroid cancer.

### The Ferroptosis-Related Eight-Gene Model Was an Independent Factor for Predicting Prognosis of Thyroid Cancer

**Figure 2:**
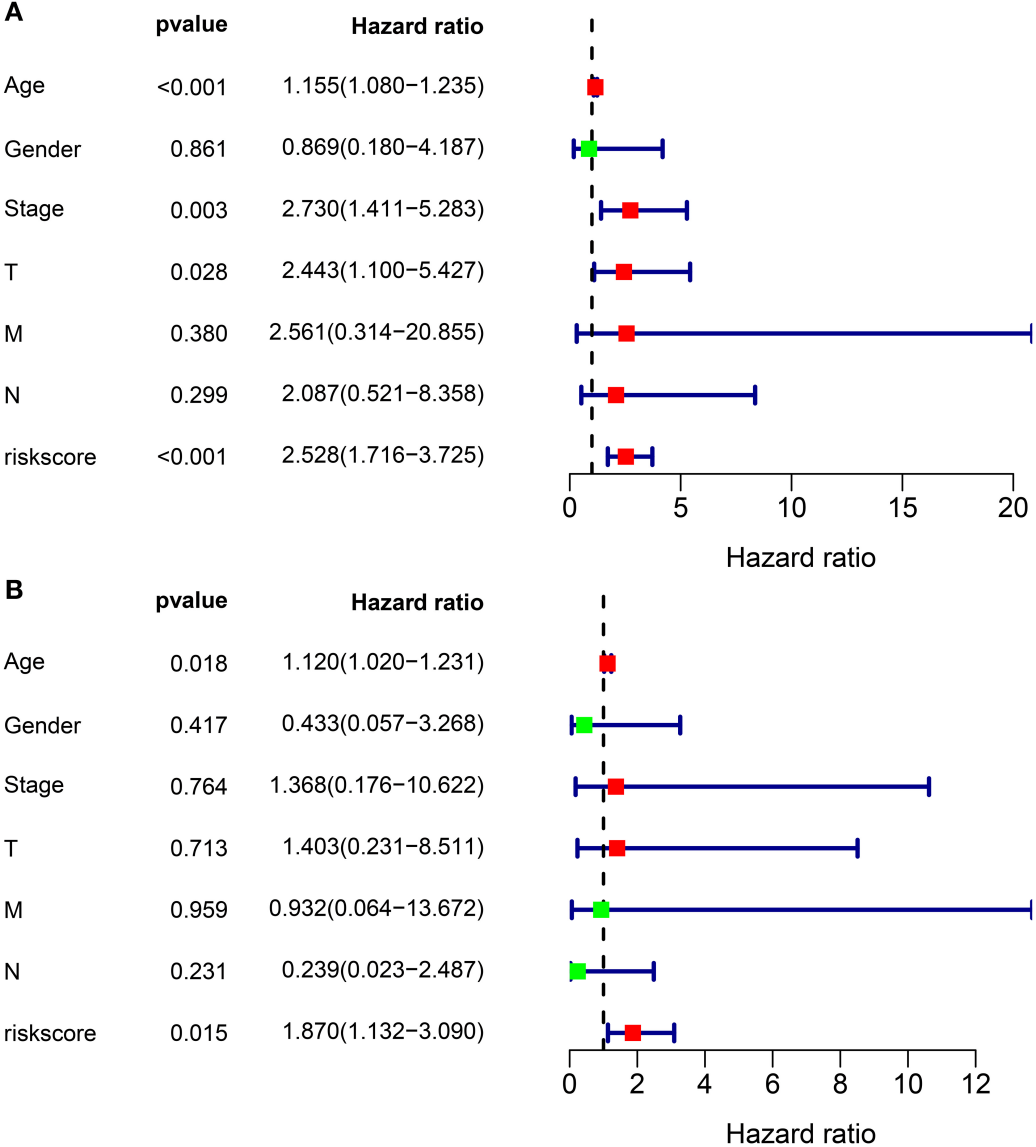
The independency of the ferroptosis-related eight-gene model for predicting the clinical outcomes for thyroid cancer. **(A)** Univariate cox regression analysis for assessment of the prognostic values of different clinicopathological characteristics (age, gender, stage, T, N, M) and the risk score. **(B)** Evaluation of the independency of the risk score and other factors for predicting the prognosis of thyroid cancer using multivariate cox regression analysis.

We further evaluated the performance of the ferroptosis-related eight-gene model for predicting the prognosis of thyroid cancer patients. Firstly, univariate cox regression analysis results showed that the risk score was a risk factor for thyroid cancer prognosis (HR: 2.528, 95% CI: 1.716–3.725, *p* < 0.001) in Figure 2A. Furthermore, age (HR: 1.155, 95% CI: 1.080–1.235, *p* < 0.001), stage (HR: 2.730, 95% CI: 1.411–5.283, *p* = 0.003), and T (HR: 2.443, 95% CI: 1.100–5.427, *p* = 0.028) were significantly associated with thyroid cancer prognosis (Figure 2A). Following multivariate cox regression analysis, the risk score was an independent risk factor for thyroid cancer (HR: 1.870, 95% CI: 1.132–3.090, *p* = 0.015; Figure 2B). Except to the risk score, age independently predicted the prognosis of thyroid cancer (HR: 1.120, 95% CI: 1.020–1.231, *p* = 0.018). Collectively, the ferroptosis-related eight-gene model was an independent prognostic factor for thyroid cancer.

### Construction of Nomogram Models for Thyroid Cancer

**Figure 3:**
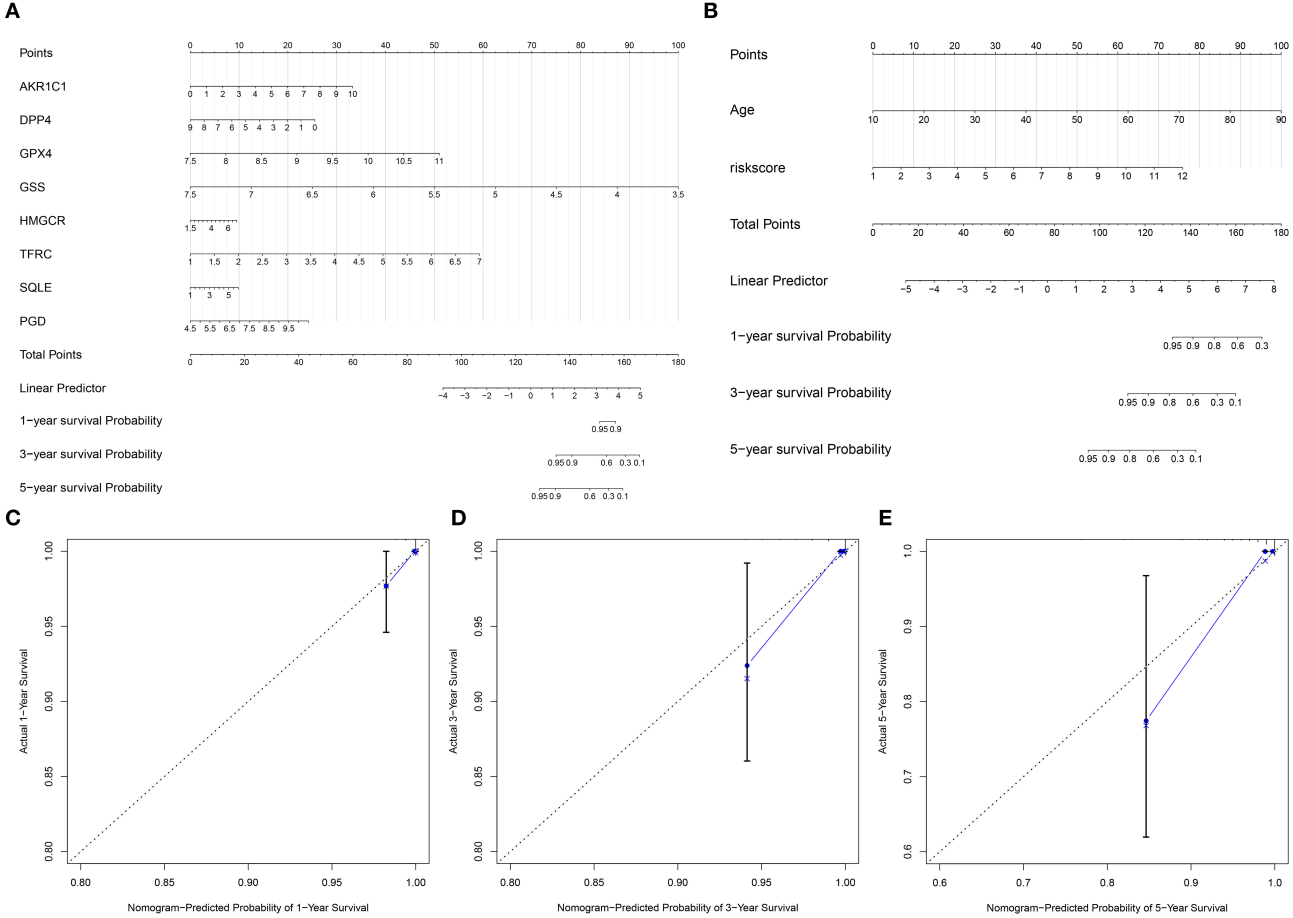
Construction of nomogram models for thyroid cancer. **(A)** A nomogram combining the eight ferroptosis-related genes. **(B)** A nomogram combining the risk score and age. **(C–E)** Calibration curves comparing the nomogram-predicted **(C)** 1-, **(D)** 3-, and **(E)** 5-year survival and actual survival.

On the basis of the eight ferroptosis-related genes that could independently predict the prognosis of thyroid cancer, a nomogram was established for predicting 1-, 3-, and 5-year survival probability of thyroid cancer patients (Figure 3A). Furthermore, by combining the two independent prognostic factors (age and risk score), we constructed a nomogram model for thyroid cancer (Figure 3B). Calibration curves confirmed that the nomogram-predicted 1- (Figure 3C), 3- (Figure 3D), and 5- (Figure 3E) year survival probability approached the actual survival.

### Stratified Analysis for the Predictive Efficacy of the Ferroptosis-Related Model

**Figure 4:**
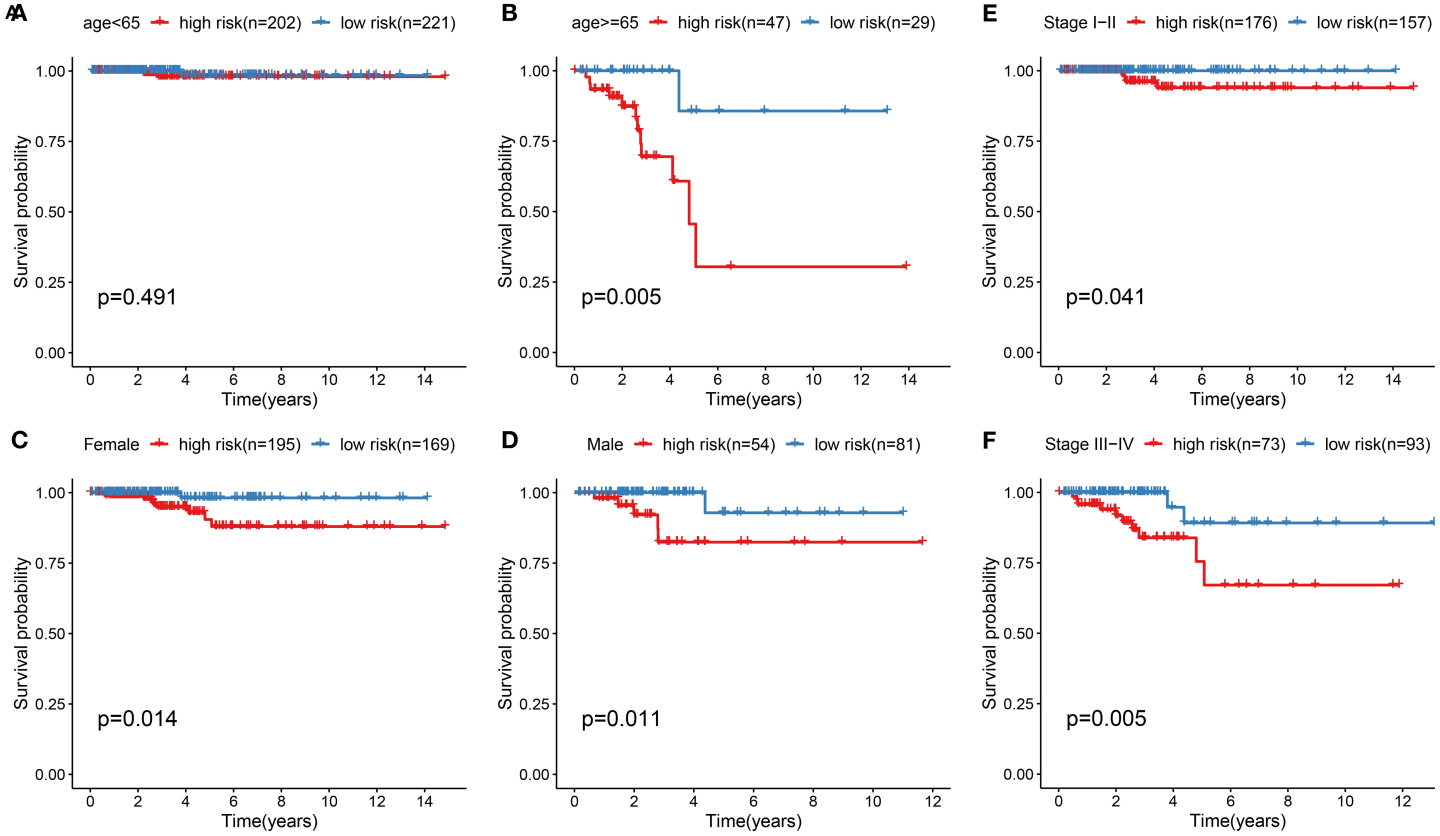
Stratified analysis for the predictive efficacy of the ferroptosis-related model among thyroid cancer samples. **(A,B)** Kaplan-Meier curves for high and low risk group in the two subgroups of age < 65 and age ≥ 65. **(C,D)** Kaplan-Meier curves for high and low risk group in the two subgroups of female and male. **(E,F)** Kaplan-Meier curves for high and low risk group in the two subgroups of stage I-II and stage III-IV.

To assess whether the ferroptosis-related eight-gene model could sensitively predict the prognosis of thyroid cancer, stratified analysis was performed. For patients with age <65, there was no significant difference in the overall survival time between high and low risk groups (Figure 4A). For patients aged >65, high risk score was indicative of the shorter survival time compared to low risk score (Figure 4B; *p* = 0.005). Both for female (Figure 4C; *p* = 0.014) and male (Figure 4D; *p* = 0.011) patients, high risk score suggested a poorer prognosis in comparison to low risk score. In Figure 4E, patients at the stage I-II in the high-risk group exhibited the less optimistic prognosis than those in the low-risk group (*p* = 0.041). Similarly, high risk score indicated the poorer clinical outcomes for patients with stage III-IV than low risk score (Figure 4F; *p* = 0.005). Collectively, the model was a sensitive prognostic marker for thyroid cancer.

### Potential Signaling Pathways for High-Risk Group

**Figure 5:**
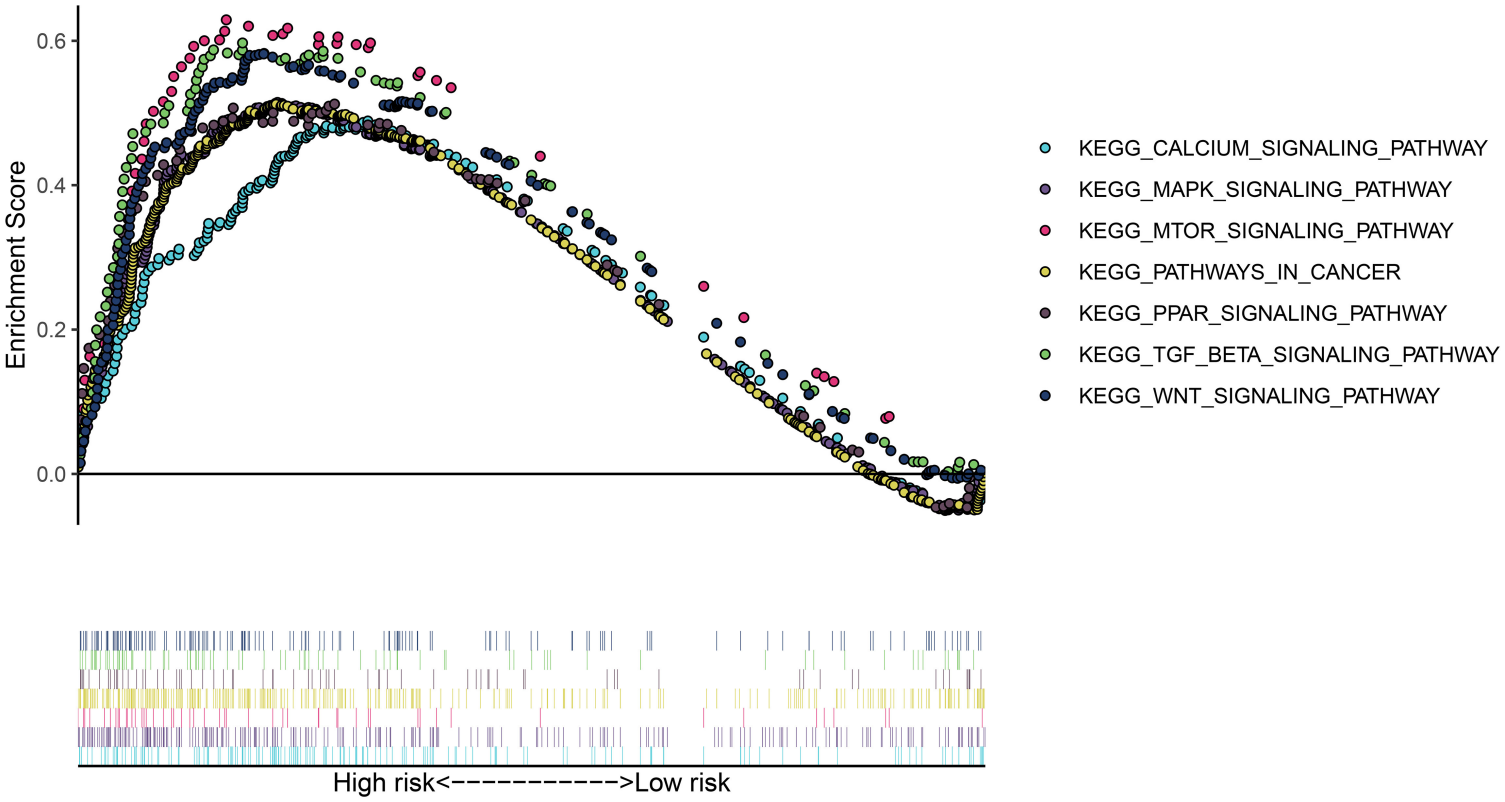
GSEA revealed the signaling pathways enriched in the high-risk group.

To uncover the potential signaling pathway in high- and low-risk groups, we presented GSEA. Our GSEA results showed that calcium signaling pathway (NES = 1.8147893 and *p* < 0.0001), MAPK signaling pathway (NES = 1.7950739 and *p* = 0.0042643924), mTOR signaling pathway (NES = 1.8197428 and *p* < 0.0001), pathways in cancer (NES = 1.7282541 and *p* = 0.008281574), PPAR signaling pathway (NES = 1.7024437 and *p* = 0.008247423), TGF-beta signaling pathway (NES = 1.8606039 and *p* = 0.0020283975), and WNT signaling pathway (NES = 1.8473499 and *p* = 0.0020876827) were significantly enriched in the high-risk group (Figure 5). However, no pathways with nominal *p*-value < 0.05 and FDR < 0.05 were found in the low-risk group, indicating that there were no pathways significantly enriched in the low-risk group.

### The Ferroptosis-Related Model Is Associated With Immune Cell Infiltration in Thyroid Cancer

**Figure 6:**
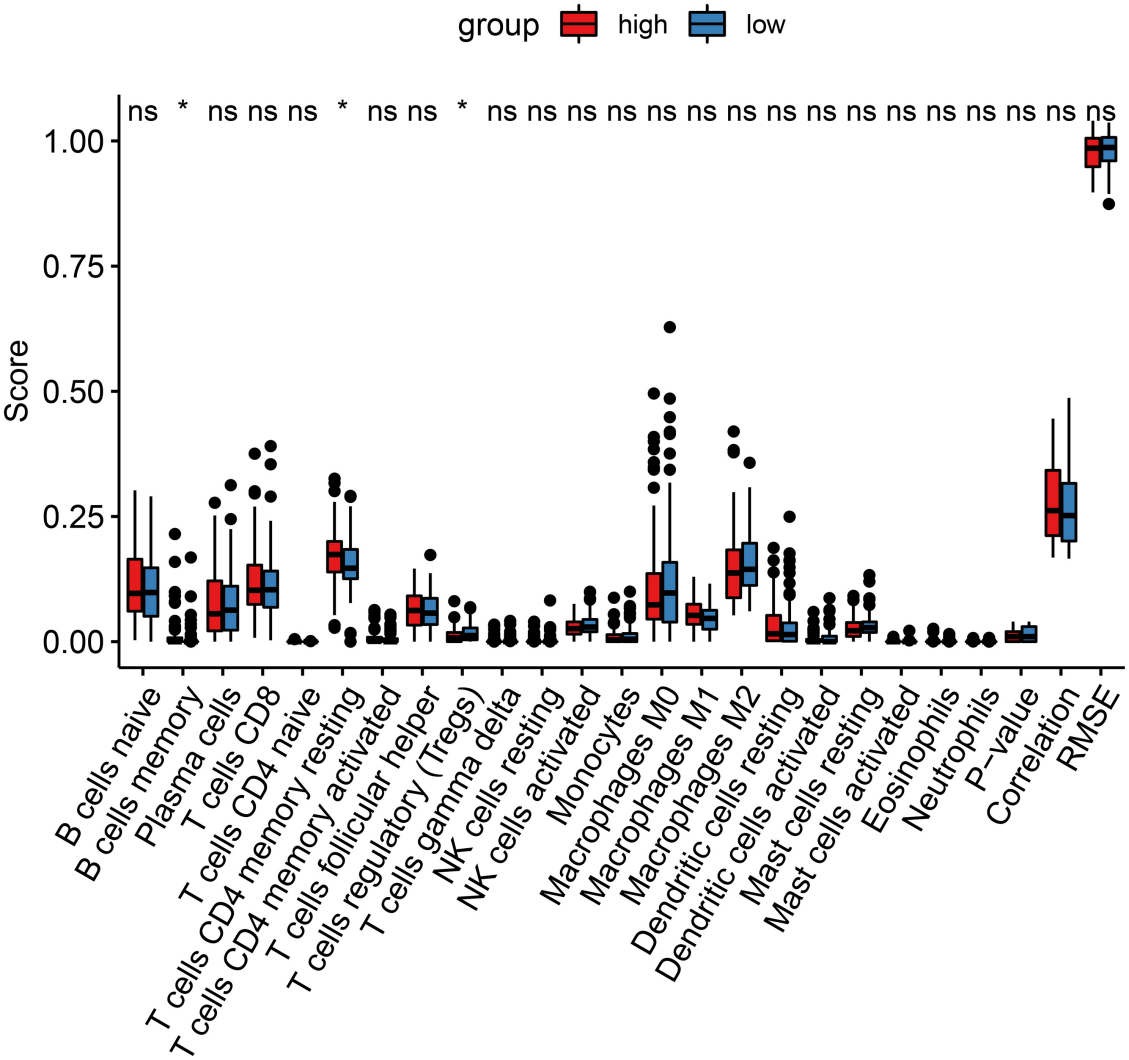
CIBERSORT identifies the association between the ferroptosis-related model and immune cell infiltration in thyroid cancer. Red, the high-risk group and blue, low-risk group. Ns, not significant; *p < 0.05.

It has been reported that ferroptosis is involved in the tumor immune microenvironment. Hein, we assessed the correlation between the ferroptosis-related risk scores and immune cell infiltrations among thyroid cancer patients using the CIBERSORT. In Figure 6, the risk score was significantly associated with the infiltration levels of B cells memory (*p* < 0.05), T cells CD4 memory resting (*p* < 0.05), and T cells regulatory (Treg; *p* < 0.05). The high-risk samples exhibited a distinctly higher infiltration level of T cells CD4 memory resting compared to the low risk samples. Lower infiltration level of Tregs was detected in the high-risk group than the low risk group.

### Validation of Eight Ferroptosis-Related Genes in Thyroid Cancer Tissues

**Figure 7:**
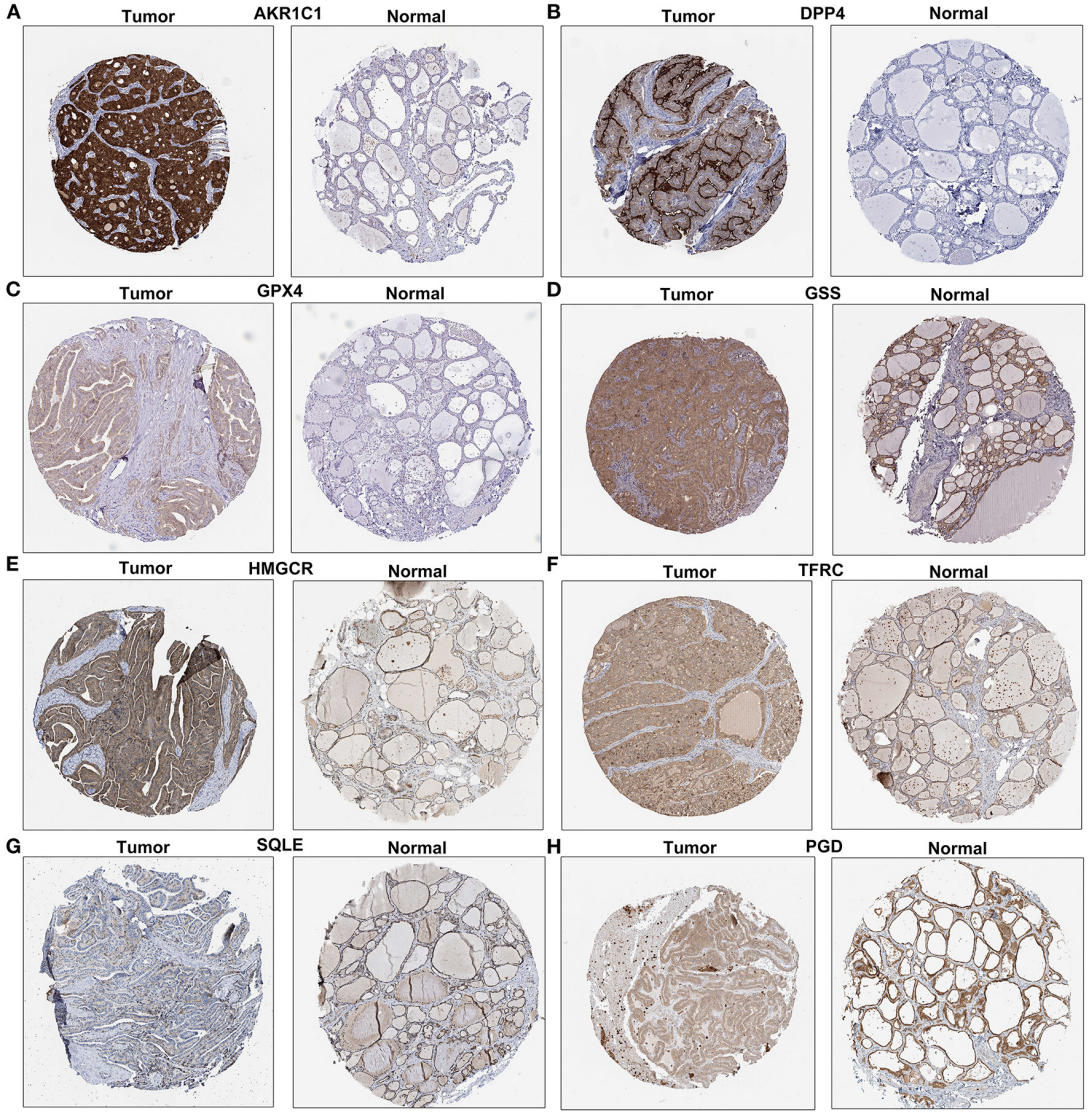
Immunohistochemistry showing the expression of **(A)** AKR1C1, **(B)** DPP4, **(C)** GPX4, **(D)** GSS, **(E)** HMGCR, **(F)** TFRC, **(G)** SQLE, and **(H)** PGD in thyroid cancer and normal tissues. Scale bar: 200 μm.

**Figure 8:**
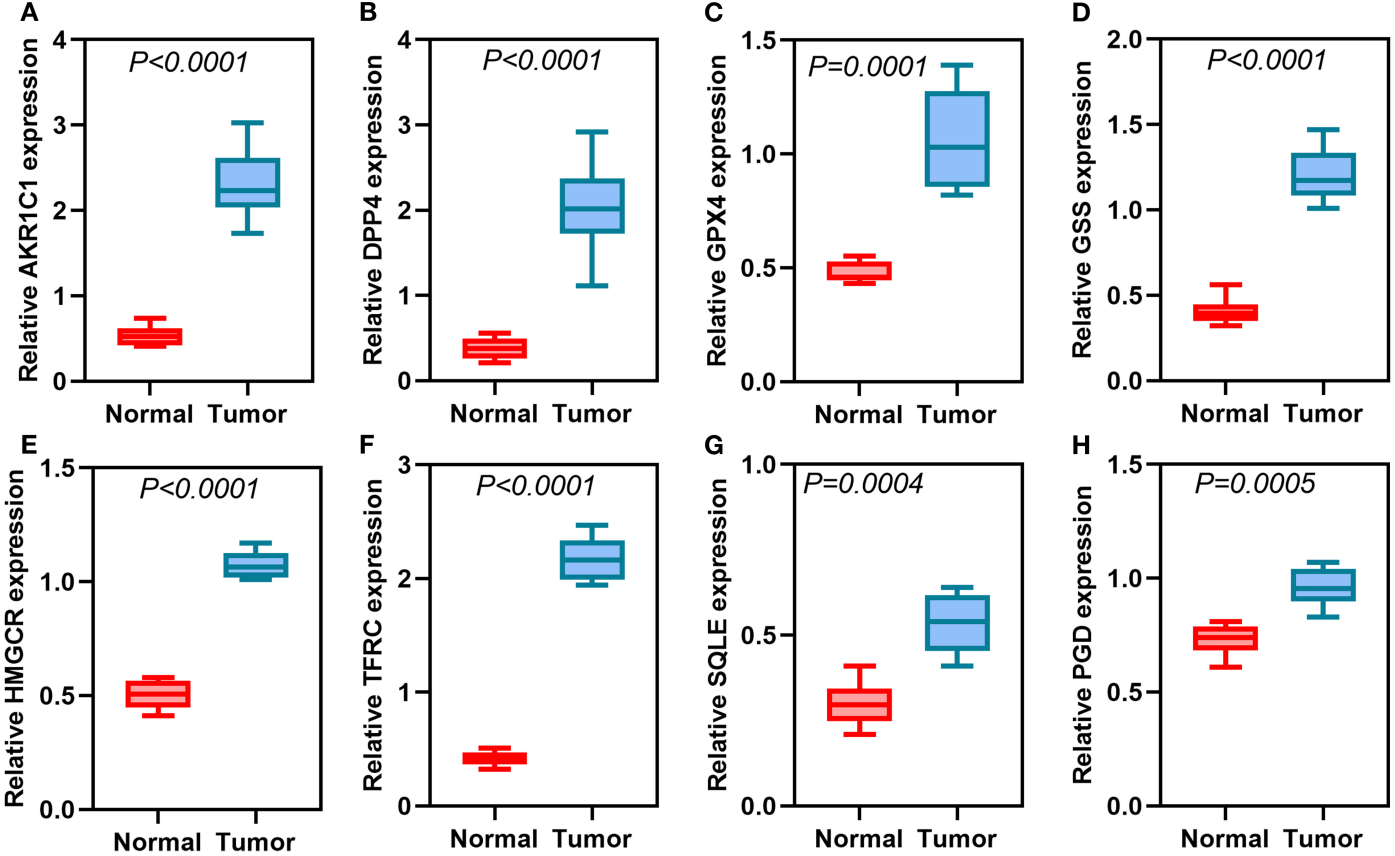
RT-qPCR detecting the mRNA expression of **(A)** AKR1C1, **(B)** DPP4, **(C)** GPX4, **(D)** GSS, **(E)** HMGCR, **(F)** TFRC, **(G)** SQLE, and **(H)** PGD in thyroid cancer and normal tissues.

We further validated the expression of the eight ferroptosis-related genes in thyroid cancer and control tissues. Immunohistochemistry results showed the expression and distribution of AKR1C1 (Figure 7A), DPP4 (Figure 7B), GPX4 (Figure 7C), GSS (Figure 7D), HMGCR (Figure 7E), TFRC (Figure 7F), SQLE (Figure 7G), and PGD (Figure 7H) in thyroid cancer and normal tissues. Furthermore, the mRNA expression levels of the eight ferroptosis-related genes were examined between thyroid cancer and normal tissues by RT-qPCR. Our data suggested that AKR1C1 (*p* < 0.0001; Figure 8A), DPP4 (*p* < 0.0001; Figure 8B), GPX4 (*p* = 0.0001; Figure 8C), GSS (*p* < 0.0001; Figure 8D), HMGCR (*p* < 0.0001; Figure 8E), TFRC (*p* < 0.0001; Figure 8F), SQLE (*p* = 0.0004; Figure 8G), and PGD (*p* = 0.0005; Figure 8H) were all significantly highly expressed in thyroid cancer tissues compared to normal tissues.

### Silencing AKR1C1 Promotes Apoptosis and Suppresses Migration and Invasion in Thyroid Cancer Cells

**Figure 9:**
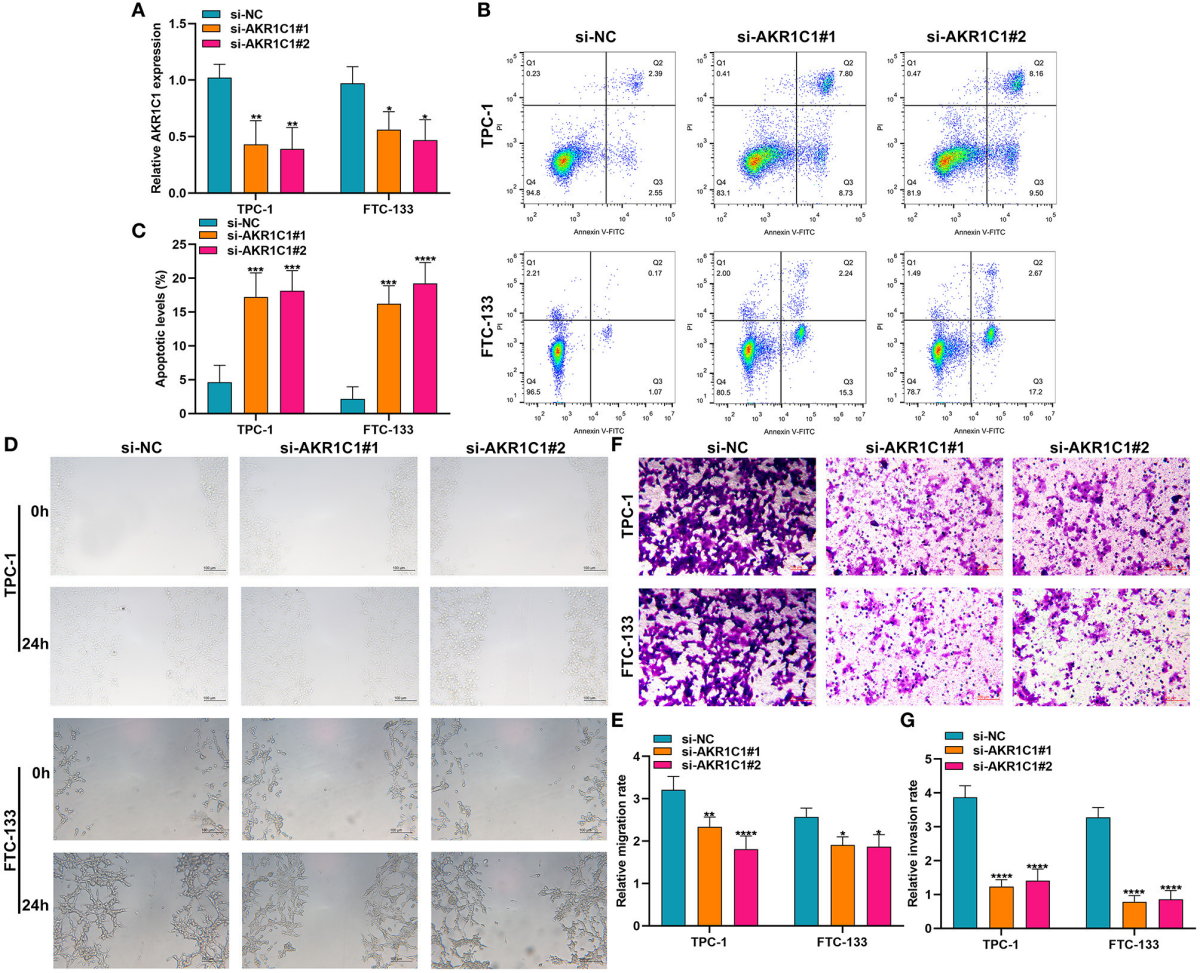
The effects of AKR1C1 knockdown on apoptosis, migration, and invasion in thyroid cancer cells. **(A)** RT-qPCR for the mRNA expression of AKR1C1 in TPC-1 and FTC-133 cells transfected with si-AKR1C1. **(B,C)** Flow cytometry detecting the apoptosis of si-AKR1C1-transfected TPC-1 and FTC-133 cells. **(D,E)** Wound healing for the migration of TPC-1 and FTC-133 cells transfected with si-AKR1C1. **(F,G)** Transwell for the invasion of TPC-1 and FTC-133 cells transfected with si-AKR1C1. *p < 0.05; **p < 0.01; ***p < 0.001; ****p < 0.0001.

Among the eight ferroptosis-related genes, we selected AKR1C1 to validate its function in thyroid cancer. AKR1C1 expression was distinctly silenced by si-AKR1C1 in TPC-1 and FTC-133 cells (Figure 9A). After silencing its expression, the apoptotic levels were significantly promoted in TPC-1 and FTC-133 cells (Figures 9B,C). Also, we found that the migrated (Figures 9D,E) and invasive (Figures 9F,G) capacities were markedly restrained by AKR1C1 knockdown in TPC-1 and FTC-133 cells. These data demonstrated that AKR1C1 might participate in thyroid cancer progression.

## Discussion

Increasing evidence emphasizes the importance of ferroptosis on the cancer therapy. Nevertheless, it remains unclear whether ferroptosis may affect the clinical outcomes for thyroid cancer patients. Thus, elucidating the molecular mechanisms and signaling pathways of ferroptosis enables us to develop novel therapeutic targets for thyroid cancer. In this study, we developed a ferroptosis-related eight gene model, which can robustly predict the prognosis of thyroid cancer.

Herein, a ferroptosis-related eight gene model was constructed for predicting the prognosis of thyroid cancer *via* LASSO Cox regression analysis. For thyroid cancer patients, high risk score was indicative of poorer prognosis than low risk score. ROC confirmed that the signature could have accurate and sensitive predictive efficacy. Multivariate cox regression analysis demonstrated that the signature can independently predict the survival for thyroid cancer. Among them, DPP4, GPX4, and GSS were protective factors for thyroid cancer prognosis. Meanwhile, AKR1C1, HMGCR, TFRC, SQLE, and PGD were risk factors for thyroid cancer prognosis. As previous studies, DPP4 is highly expressed in thyroid cancer tissues, which has been considered as a prognostic factor (15). It can induce proliferative, invasive, as well as migrated capacities for thyroid cancer (16). GPX4 is an important regulator of ferroptosis for cancer cells (17). Depletion of GPX4 may induce ferroptosis, which can sensitize cells to ferroptosis (18). AKR1C1 can activate STAT3, thereby promoting lung cancer metastasis (19). Furthermore, it mediates cisplatin-resistance for head and neck squamous cell carcinoma *via* STAT1/3 pathways (20). Targeting HMGCR may suppress the invasive ability of thyroid cancer cells (21). It has been reported that YAP can facilitate ferroptosis *via* up-regulation of a ferroptosis regulator TFRC in cancer cells (22). Moreover, TFRC induces epithelial ovarian cancer cell proliferation as well as metastases by up-regulating AXIN2 (23). SQLE has become a pharmaceutical target for various cancers. For example, it may drive non-alcoholic fatty liver disease-induced hepatocellular carcinoma (24). Additionally, SQLE expression can predict the fatality rate of prostate cancer (25). However, the functions of these eight ferroptosis-related genes remains to be clarified in thyroid cancer. Our immunohistochemistry and qRT-PCR confirmed that these eight genes were all activated in thyroid cancer. This study may offer novel clues for exploring the biological roles and clinical significance of these ferroptosis-related genes. Our multivariate cox regression analysis suggested that age at diagnosis was an independent risk factor for thyroid cancer. Consistently, age has been commonly applied as a risk factor for stratifying the prognosis of thyroid cancer (26). Among adults aged over 65 years old, the incidence of thyroid cancer is the highest (27). Our stratified analysis revealed that the ferroptosis-related eight gene model could accurately predict the clinical outcomes for patients with >65 years old. Moreover, regardless of whether it was a male or female patient, high risk score implied a worse prognosis. For patients with stage I-II or III-IV, high risk score was indicative of shorter survival time. Hence, the signature could accurately predict the clinical outcomes for thyroid cancer. In order to expand the clinical application of this model, we combined it with age to construct a nomogram that could be easy to calculate the expected survival rate for an individual patient. Calibration curves confirmed that the nomogram-predicted 1-, 3-, and 5-year survival time was close to the actual survival time. This suggested that the nomogram had the potential as a clinically predictive tool for thyroid cancer prognosis.

Various signaling pathways participate in regulating the process of ferroptosis. Herein, our data suggested that MAPK, mTOR, pathways in cancer, PPAR, TGF-beta, and WNT signaling pathways were enriched in the high-risk group. MAPK pathway can be activated in ferroptosis cells (28). In turn, blockage of MAPK pathway also protects cells from ferroptosis (29). The activation of MAPK pathways can lead to the occurrence and progression of thyroid cancer (30). Ferroptosis-induced ROS accumulation could inactivate MAPK pathway to kill thyroid cancer cells (30). Four targeted therapies (Sorafenib, Lenvatinib, Vandetanib, and Cabozantinib) have been approved for treating thyroid cancer patients resistant to standard therapy, which can have the effects *via* blockage of MAPK pathway (31). Hence, their capacity to prolong the survival time of patients remain to be limited because of poor efficacies and other molecular interventions such as ferroptosis (31). mTOR pathway can mediate cellular proliferative capacities and the uptake of iodine for thyroid cells (32). Recently, mTOR pathway has been confirmed to be involved in mediating ferroptosis in different cancers such as colorectal cancer (33). Activation of mTOR transduction may protect cancer cells from oxidative stress and ferroptosis (34). PPARα promotes ferroptosis by mediating lipid remodeling in cancer cells (35). In WNT pathway, Frizzled-7 is sensitive to ferroptosis for platinum-tolerant ovarian cancer cells (36). Taken together, our results indicated that various pathways could be involved in ferroptosis for thyroid cancer. The roles of these pathways in ferroptosis should be assessed by more experiments.

Many thyroid cancer patients who are not accompanied by thyroiditis, immune infiltrations are found following surgery, suggesting that changes in the immune microenvironment is related to the progression of thyroid cancer (37). Our data demonstrated that the model was significantly associated with the infiltration of B cells memory, T cells CD4 memory resting and Tregs. Further analysis should be presented to probe into the associations between the ferroptosis-related model and the tumor microenvironment. A larger cohort should be required to verify the predictive values of the ferroptosis-related eight gene model for thyroid cancer.

## Conclusion

Collectively, we constructed a ferroptosis-related eight gene model that exhibited a well predictive performance. The model had a significant association with the tumor immune microenvironment. The nomogram combining the model and age could provide new possibilities for individualized therapy of thyroid cancer patients. Hence, ferroptosis could be promising therapeutic targets.

## Data Availability Statement

The datasets presented in this study can be found in online repositories. The names of the repository/repositories and accession number(s) can be found in the article/Supplementary Material.

## Ethics Statement

The studies involving human participants were reviewed and approved by the Ethics Committee of Linyi Central Hospital (2019019). The patients/participants provided their written informed consent to participate in this study.

## Author Contributions

PH conceived and designed the study. MG, JN, and AT conducted most of the experiments and data analysis, and wrote the manuscript. YD, FX, and FL participated in collecting data and helped to draft the manuscript. All authors reviewed and approved the manuscript.

## Conflict of Interest

The authors declare that the research was conducted in the absence of any commercial or financial relationships that could be construed as a potential conflict of interest.

## Supplementary Material

Supplementary Table 1 A list of 60 ferroptosis-related genes.

## Abbreviations

RNA-seq: RNA sequencing
TCGA: The Cancer Genome Atlas
LASSO: least absolute shrinkage and selection operator
ROC: Receiver Operating Characteristic
PCA: Principal Component Analysis
GSEA: Gene Set Enrichment Analysis
FDR: false discovery rate
qRT-PCR: quantitative real-time PCR
HR: hazard ratio
CI: confidence interval
AUC: area under the curve.
